# Mucopolysaccharidosis type III (Sanfilippo syndrome) and misdiagnosis of idiopathic developmental delay, attention deficit/hyperactivity disorder or autism spectrum disorder

**DOI:** 10.1111/apa.12169

**Published:** 2013-02-06

**Authors:** Frits A Wijburg, Grzegorz Węgrzyn, Barbara K Burton, Anna Tylki-Szymańska

**Affiliations:** 1 Department of Paediatrics, Academic Medical CentreAmsterdam, The Netherlands; 2 Department of Molecular Biology, University of GdańskGdańsk, Poland; 3 Division of Genetics, Birth Defects and Metabolism, Ann & Robert H. Lurie Children's Hospital of ChicagoChicago, IL, USA; 4 Department of Metabolic Diseases, The Children's Memorial Health InstituteWarsaw, Poland

**Keywords:** Attention deficit/hyperactivity disorder, Autism spectrum disorder, Developmental disabilities, Mucopolysaccharidosis type III, Speech/language disorders

## Abstract

Mucopolysaccharidosis III is a rare genetic disease characterized by progressive cognitive decline and severe hyperactivity that does not respond to stimulants. Somatic features are relatively mild. Patients are often initially misdiagnosed as having idiopathic developmental delay, attention deficit/hyperactivity disorder and/or autism spectrum disorders, putting them at risk for unnecessary testing and treatments.

**Conclusion:** Children with developmental or speech delay, especially those with a characteristic somatic feature or behavioural abnormalities, should be screened for MPS III.

## Introduction

The mucopolysaccharidoses (MPSs) are a group of seven inherited metabolic disorders within the larger lysosomal storage disease (LSD) family 1. As with most of the LSDs, each of the MPSs is characterized by the absence or deficiency of a specific lysosomal enzyme that leads to the accumulation of undigested or partially digested macromolecules within lysosomes. In the MPSs, the accumulation of glycosaminoglycans (GAGs) – long unbranched polysaccharides that play an essential role in connective tissue biology and cellular crosstalk – produces progressive cellular damage that results in multisystemic disease.

Of the seven MPSs, mucopolysaccharidosis type III (MPS III or Sanfilippo syndrome) is the most common 2. MPS III is characterized by early-onset developmental delay and/or speech delay after an initial period of normal development. This is followed by progressive cognitive decline, behavioural abnormalities and severe hyperactivity that does not respond to stimulant medication. Somatic features are relatively mild. Young children with MPS III are often misdiagnosed as having idiopathic developmental/speech delay, attention deficit/hyperactivity disorder (ADHD) and/or autism spectrum disorders 1,3.

Mucopolysaccharidosis III is composed of four different subtypes: type A (OMIM #252900), type B (OMIM #252920), type C (OMIM #252930) and type D (OMIM #252940). Each subtype is caused by a deficiency in a different enzyme in the catabolic pathway for heparan sulfate, a type of GAG. All four subtypes are inherited in an autosomal recessive pattern. The incidence of the subtypes has a very uneven geographic distribution. Together, the reported incidence of all subtypes of MPS III varies between 0.28 and 4.1 per 100 000 live births, with types A and B being more common than types C and D 3.

**Key notes**

- Mucopolysaccharidosis III (Sanfilippo syndrome) is a rare, progressive genetic disease that presents with onset of developmental or speech delay after a period of normal development, followed by severe behaviour problems and hyperactivity.
- Common misdiagnoses are idiopathic developmental/speech delay, attention deficit/hyperactivity disorder or autism spectrum disorders.
- Children with developmental delay, especially coupled with a characteristic somatic feature or behavioural abnormality, should be screened for MPS III.

Diagnostic delay in the MPS III population is very common, particularly in patients with a slowly progressing, or attenuated, phenotype. In a series of Dutch studies, it was found that the average delay between the first presenting sign/symptom of the disease and correct diagnosis was between 1.5 and 9 years, depending upon the subtype 4–7. Historically, a long diagnostic delay has not been considered to be an acute problem for patients with MPS III because treatment options have been limited to supportive care. However, there are now a number of therapeutics in development that are aimed at altering the underlying pathophysiology. Human clinical trials are in progress for intrathecal enzyme replacement therapy (ERT) and substrate reduction therapy, while gene therapy and chemical chaperone therapy are being studied in animal models (reviewed in 8). It is thought that the best chance for optimum patient outcome will occur when these types of therapies are initiated before extensive neurological damage has begun; thus, early recognition and diagnosis are now seen as key for patients with MPS III 8. In the absence of newborn screening, paediatricians, paediatric specialists and other paediatric clinicians are the major drivers of this effort because they are the providers most likely to see patients in the early stages of MPS III. Here, we describe the signs, symptoms and patient history that should trigger suspicion of MPS III and then focus on the practicalities of the diagnostic process and referral. Even after referring patients to a metabolic disease specialist, the paediatrician retains a major role in the multidisciplinary care team.

## Clinical Picture

As with all of the MPSs, patients with MPS III can present with signs and symptoms that may fall at any point along a spectrum of severity. For ease of description, phenotypes are generally described as ‘severe’ and ‘attenuated’, with distinct natural histories. For all phenotypes, MPS III is a progressive disease with three phases that begin after a period of apparently normal development (Table 1). As the severe phenotype is usually more common, we shall describe its natural history first. In the first phase, generally starting between the ages of 1 and 3 years, a slowing or plateauing of cognitive development becomes apparent; often speech is more noticeably affected than other cognitive functions 9. Motor development usually progresses normally during this stage. Characteristic somatic signs and symptoms (described below) may emerge. The second phase starts at approximately 3–4 years of age and is characterized by progressive cognitive deterioration and the emergence of behavioural difficulties and sleep disturbances. Behavioural difficulties, including hyperactivity, impulsivity, obstinacy, anxious behaviours and autistic-like behaviours, worsen over time and can become extreme 10. The third stage begins, usually in the teenage years, with the onset of severe dementia and motor function decline. Behavioural problems slowly disappear as patients lose locomotion. Swallowing difficulties and spasticity emerge. Patients eventually regress to a fully bedridden and vegetative state, and they usually die at the end of the second or beginning of the third decade of life 4–7,11,12.

**Table 1:** The three phases of mucopolysaccharidosis type III (MPS III) and associated signs and symptoms

Phase*	Signs/Symptoms†
Presymptomatic	Apparently normal development
Phase 1	Neurocognitive
	Developmental delay
	Speech delay
	Somatic
	Mild facial dysmorphism (can be very subtle)
	Frequent ear infections
	Frequent upper respiratory infections
	Cardiac valve disease
	Hernia (umbilical, inguinal)
	Hepatomegaly
	Diarrhoea
Phase 2	Neurocognitive
	Progressive cognitive decline/mental retardation
	Decline in speech/lack of speech
	Behavioural disturbances
	Hyperactivity
	Impulsivity
	Aggression
	Restlessness
	Anxious behaviour
	Compulsive behaviour
	Autistic-like behaviour
	Decline in motor skills
	Seizures
	Somatic (those in phase 1, plus the following)
	Hearing loss
	Orthopaedic manifestations
	Scoliosis
	Kyphosis
	Lumbar lordosis
	Hip dysplasia and pain
	Carpal tunnel syndrome
	Trigger digits
	Joint contractures
Phase 3	Neurocognitive
	Profound mental retardation progressing to vegetative state
	Lack of speech or communication
	Behavioural disturbances cease
	Difficulty swallowing progressing to inability to swallow
	Spasticity
	Seizures
	Somatic
	Those in phases 1 and 2

* The timing of the disease course in attenuated patients is more variable than that seen in severe patients, but progression through these phases is common to all MPS III patients.

† Not all signs and symptoms may be present in any individual patient.

With the attenuated phenotype, a more gradual disease progression with longer survival is seen 5,13,14. The first phase begins as a mild developmental and/or speech delay around the age of 4 years. As with the severe phenotype, characteristic somatic manifestations (described below) can appear at this stage. For the attenuated phenotype, it is the second stage that slows down considerably. Mild cognitive impairment may remain stable into the teenage years or even adulthood before progressing. Behaviour problems, similar to those seen in severe patients, do emerge in attenuated patients, but may emerge later or be more manageable in degree. The third stage and death usually occur in the fourth to sixth decade of life, with cases of survival up to nearly 70 years of age being reported 5,13,14.

Patients with attenuated disease may easily remain undiagnosed until adulthood, as early diagnosis in this population is particularly challenging. In one study of all MPS IIIB patients ever diagnosed in the Netherlands, 33 of 52 patients displayed an attenuated phenotype 5. This is a much higher percentage of attenuated patients than is generally seen in studies of the phenotypic distribution in MPS III A, C and D 1,4,6,7,9. Nearly all of these attenuated patients (32/33) survived to adulthood. Although the first clinical sign of developmental delay was observed at a median age of 4 years, loss of speech was not reported until a median age of 35 years (range, 8–68 years) and the ability to walk at a median age of 42.5 years (range, 18–68 years). The median age at diagnosis in this group of patients was 28 years. These older patients were reported to be able to function with a stable intellectual disability for many years. Indeed, cases have been reported in the literature describing elderly patients with dementia or behavioural disturbances who are not ultimately diagnosed with MPS III until their sixth to eighth decade of life 13. Based on this, it seems likely that many patients with an attenuated phenotype are never diagnosed, although the exact percentage of patients in this situation cannot be known unless systematic newborn screening programmes are put into place.

### Neurocognitive manifestations

Unlike the other MPSs that present with extensive somatic involvement, patients with MPS III typically present with mainly cognitive and neurological signs and symptoms 1,3. Here we will focus on those manifestations seen in phases 1 and 2 in order to facilitate early diagnosis. The first neurocognitive complaint, seen in phase 1, is usually developmental delay, often in speech. In a large natural history study of 107 patients in France, speech delay at diagnosis was seen in 93%, 88%, 92% and 66% of patients with MPS III types A, B, C and D, respectively 15. An isolated speech delay with normal development in other areas is not uncommon, leading to the misdiagnosis of idiopathic speech delay. Other patients present with a more global developmental delay, which can lead to misdiagnoses of autism spectrum disorder or idiopathic developmental delay.

Following the developmental and/or speech delay, behavioural difficulties and sleep disturbances emerge in phase 2. These can include severe hyperactivity, uncontrolled impulsivity, autistic-like behaviour and excessive anxiety 16. In practice, it can be difficult for the clinician to distinguish MPS III behavioural difficulties from ADHD or autism spectrum disorders. In MPS III, hyperactivity may be marked, with extreme restless behaviour, temper tantrums and crying or laughing fits. Impulsivity may be such that patients may have little to no regard for their own safety. Parents may report the need for constant supervision. One characteristic feature of the behaviour is that it does not respond or responds poorly to standard stimulant medications and does not respond to behaviour-based interventions. Extreme difficulty in falling asleep and frequent night waking further complicate behavioural problems 17. Over 90% of children with MPS III are reported to have sleep disturbances, which can be debilitating for the family. Patients may wander, sing, shout or talk throughout the night. Some patients have been reported to sleep for as little as 2 h per night.

### Somatic manifestations

**Figure 1:**
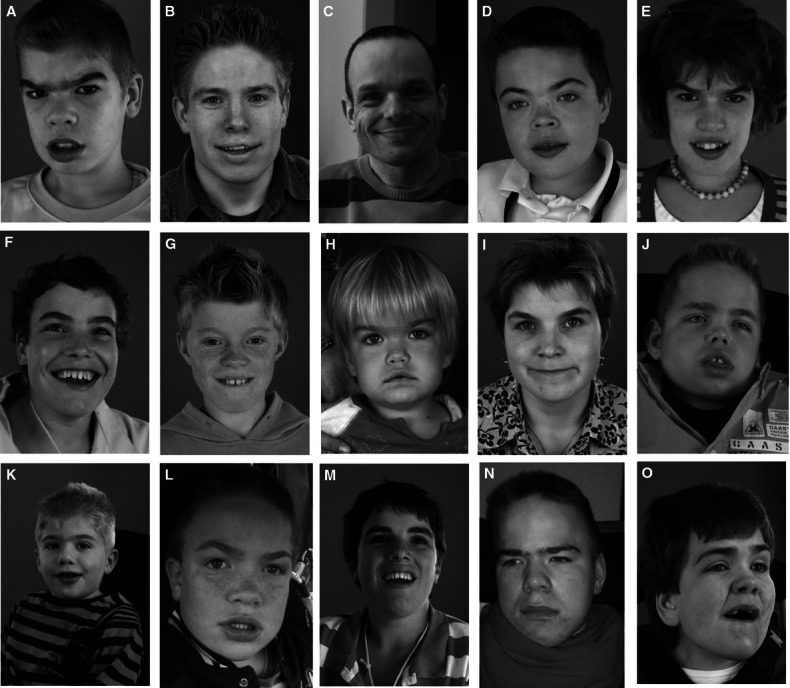
Gallery of facial images from patients of various ages with MPS III. Not all affected patients have discernible facial dysmorphisms. Because of the variability in disease progression, early diagnosis does not always mean diagnosis in a young child; here we represent a variety of ages. The sex, MPS III subtype and age of the patients are as follows: (a) male, MPS IIIC, 10 years; (b) male, MPS IIIB, 21 years; (c) male, MPS IIIA, 43 years; (d) male, MPS IIIC, 10 years; (e) female, MPS IIIC, 13 years; (f) female, MPS IIIB, 18 years; (g) male, MPS IIIB, 11 years; (h) female, MPS IIIC, 4 years; (i) female, MPS IIIA, 20 years; (j) male, MPS IIIA, 10 years; (k) male, MPS IIIA, 6 years; (l) male, MPS IIIA, 11 years; (m) female, MPS IIIB, 20 years; (n) male, MPS IIIA, 12 years; (o) female, MPS IIIA, 14 years. MPS III, mucopolysaccharidosis type III.

Somatic symptoms are heterogeneous in the MPS III population and can be much more subtle than those seen in the other MPS disorders 3,6. Although facial dysmorphisms are easily discernible in MPS I-Hurler or MPS II (Hunter syndrome), patients with MPS III often have only mildly coarse facial features (Fig. 1). These may be more obvious in younger patients in phase 1 or early phase 2 13. If coarse facies are present, there may be a dolichocephalic skull shape with a short forehead, prominent eyebrows, an everted and thick lower lip and an upturned upper lip with a protruding philtrum. Hirsutism, a low hair line and very coarse, stiff hair may also be present. However, a lack of overt facial dysmorphisms should not rule out the disorder 3,13. Similarly, although certain other LSDs are associated with a high incidence of hepatomegaly and splenomegaly, only about half of patients with MPS III display hepatomegaly, and very few have splenomegaly 4–7. Normal liver and spleen size thus do not rule out the diagnosis. Recurrent ear and respiratory infections are common in the MPS III population but unfortunately are nonspecific in the paediatric population 4–7. Orthopaedic manifestations (scoliosis, kyphosis, lumbar lordosis, hip dysplasia and pain, carpal tunnel syndrome, and trigger digits) are a feature of MPS III, although these tend to appear later in the second phase of the disease and only in a minority of patients 18.

### Early recognition of MPS III

**Figure 2:**
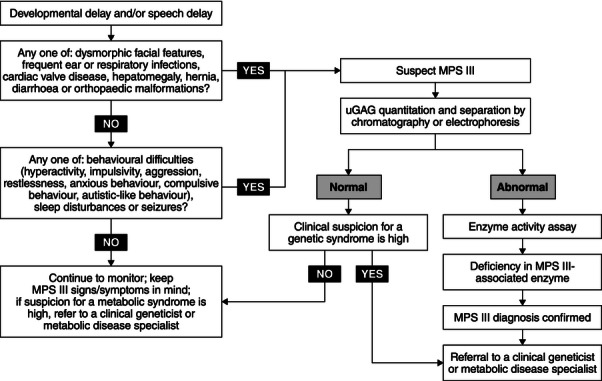
Diagnostic algorithm for mucopolysaccharidosis (MPS) III. MPS III, mucopolysaccharidosis type III; uGAG, urinary glycosaminoglycan.

It is clear that MPS III is a diagnostic challenge, particularly in the early stages and in the absence of a family history of the disease. The clinician must see the whole child and connect the various signs and symptoms together into a pattern suggesting a metabolic disorder (Table 1). In phase 1, look for a young child who presents with developmental and/or speech delay with a characteristic somatic sign or symptom (e.g. mild facial dysmorphisms, frequent ear or respiratory infections, cardiac valve disease, hernia, hepatomegaly or diarrhoea). If one of these somatic features is present, initiating diagnostic testing is recommended (Fig. 2). In fact, we routinely test children using the urinary glycosaminoglycan (uGAG) screening assay (described below) who present with developmental delay/speech delay and no other somatic signs and symptoms. This is not unreasonable because the uGAG assay is not expensive and is not invasive. Such children can be easily misdiagnosed with autism or pervasive developmental disorder and may be subjected to more invasive testing, dietary restrictions or even unproven alternative therapies that eventually prove unnecessary or perhaps harmful.

In phase 2, the presence of developmental delay is joined by a host of hard-to-manage behavioural difficulties and sleep disturbances. A lack of response to standard stimulant medications for ADHD or to behaviour-based interventions is a key diagnostic clue in this phase. Any of these neurocognitive signs in combination, or in combination with an MPS III somatic feature, should trigger clinical suspicion of the disease and start the diagnostic process (Fig. 2). When in doubt, test! This can spare the patient and family unnecessary tests, exposure to inappropriate medications and a long, difficult journey to diagnosis.

## Biochemical Testing for MPS III

When choosing a laboratory for biochemical testing for MPS III, it is important to keep in mind that not all laboratories are able to perform the necessary assays, especially those for genetic and prenatal testing. The units used to describe test results and normal ranges can vary between laboratories, which is particularly true for uGAG testing. Ensure that the laboratory used is accredited and experienced in the desired analyses; listings such as GeneTests may be helpful.

### uGAG excretion

Mucopolysaccharidosis III is characterized by an inability to catabolize heparan sulfate, leading to the excretion of this molecule in the urine 1. A uGAG assay is therefore the usual first step in making a biochemical diagnosis, as it is noninvasive and convenient – uGAGs stay stable at room temperature for up to 10 days, so urine samples do not need to be frozen for transport to the laboratory 19. Both semi-quantitative assays (e.g. Berry spot, Ames spot) and quantitative assays (e.g. dimethylmethylene blue, alcian blue, and azure A and B) are in clinical use, although the semi-quantitative assays are prone to false-negatives and false-positives and are better avoided 20,21. The quantitative assays can detect the absence or presence of excess GAGs in the urine, although they do not allow for the diagnosis of a particular MPS subtype. Thus, all samples should be analysed via electrophoresis or thin layer chromatography to identify the specific pattern of GAG excretion 22,23.

Although uGAG testing is easy to perform, there are several caveats to keep in mind. First, heterozygous carriers of MPS III cannot be diagnosed using uGAG assays because they have normal GAG excretion. Second, among the MPSs, patients with MPS III may have comparatively lower levels of uGAGs and can receive a false-negative result 24. In one study, the uGAG levels in 33 of 58 patients with MPS III were found to overlap substantially with the levels seen in controls 25. Thus, it is important to ensure that urine samples are not dilute. Collecting three consecutive first-morning urine samples on three consecutive days is a useful practice. Even when using concentrated first-morning samples, however, a fraction of patients with MPS III still have apparently normal uGAG levels, so a negative uGAG test *does not* rule out the diagnosis. Third, false-positives may occur with a uGAG assessment, particularly if electrophoresis is used to identify the accumulated GAG species. Heparin migrates in the same position as heparan sulfate on electrophoresis, leading to a false-positive if the urine sample is put in a heparinized tube. With these caveats in mind, uGAG screening is a useful first step as it is simple, noninvasive and inexpensive. In the future, technology such as high-performance liquid chromatography/mass spectrometry may be used to reduce the time needed to perform the assay and the false-negative rate; such approaches are under investigation 26–29.

### Enzyme activity assays

Enzyme activity assays are the gold standard used to confirm the diagnosis and to determine the subtype. Enzyme activity can be measured in leucocytes or cultured fibroblasts, and all four enzymes for the subtypes of MPS III can be assayed 30–33. Enzyme activity levels in chorionic villi and amniotic fluid cells can also be measured for prenatal diagnosis 34,35. Very low or absent activity for one of the enzymes is diagnostic of MPS III of the applicable subtype. In the case of MPS IIIA and IIID, which each result from deficiency of a specific sulfatase, a second sulfatase should also be measured in the patient's sample to rule out multiple sulfatase deficiency, an LSD that affects the entire sulfatase family 36. As with the uGAG assays, enzyme activity assays are not useful for the identification of heterozygous carriers because of overlap between the activity ranges of carriers and normal controls 37. Enzyme activity assays utilizing dried blood spots on filter paper are under development for many LSDs, including MPS III, and may allow for newborn screening in the future 38.

There has been much interest in determining the prognostic value of residual enzyme activity level for phenotypic severity. It has been demonstrated that residual enzyme activity levels alone do not reliably correlate with disease phenotype (i.e. severe vs. attenuated) and should not be used for this purpose 39. This must be emphasized to families. Preliminary research suggests that phenotype prediction may be made from a simultaneous analysis of residual enzyme activity and the efficiency of GAG synthesis in patients' cells 39. However, the latter parameter is not commonly tested in clinical practice and can be measured in only a small number of diagnostic laboratories, so such analyses remain experimental.

### Genetic testing

Molecular genetic testing can be offered to every family with a child affected by MPS III. It is the only way to identify heterozygous carriers of the disease, allowing for informed genetic counselling and family planning decision-making. Molecular genetic testing in chorionic villi or amniotic fluid cells is available at some centres and can be offered to couples with a family history of MPS III as a method of prenatal diagnosis in subsequent pregnancies. Preimplantation genetic diagnosis has been reported for a few families for MPS I, II and IV, but has not been reported for MPS III 40–42. Certainly the ethical considerations surrounding genetic testing for a disease for which there is no currently approved therapy should be discussed with the family before testing is undertaken. These issues have been reviewed at length elsewhere, particularly in regard to the identification of presymptomatic patients if newborn screening were to become a reality 43,44.

There are numerous reported mutations for the four disease-causing genes: 115 for sulfamidase (MPS IIIA), 134 for a-*N*-acetylglucosaminidase (MPS IIIB), 54 for acetyl-CoA:a-glucosaminide *N*-acetyltransferase (MPS IIIC) and 23 for *N*-acetylglucosamine-6-sulfate sulfatase (MPS IIID) [Bibr b45]. Although many researchers have attempted genotype–phenotype correlations for all the subtypes of MPS III, these have proven difficult because of the immense allelic heterogeneity and because of polymorphisms that may influence the clinical phenotype by modifying the residual activity of mutant enzyme 46. Certain more commonly occurring alleles have been associated with a particular phenotype, and this information can be communicated to patients and families when applicable.

## After the Diagnosis: Referral and Treatment Options

Patients with a confirmed diagnosis of MPS III, or those for whom there is a strong clinical suspicion, should be promptly referred to a metabolic disease specialist. Once the referral has been made, the paediatrician maintains a key role in overseeing the management of these patients because parents and caregivers easily become overwhelmed with the demands of care. The child may need to be seen by multiple specialists, including specialists in cardiology, neurodevelopment, ophthalmology, orthopaedics, otorhinolaryngology, psychiatry and pulmonology. In addition, support services such as physiotherapy, occupational therapy, speech therapy, audiology and behavioural therapy are usually required. The managing paediatrician/primary care physician may also encourage the family to seek out additional support through patient/family support organizations.

Current treatment options are limited to supportive care. Hematopoietic stem cell transplantation (HSCT) with bone marrow cells has been attempted in this population based upon positive outcomes in patients with MPS I, MPS VI and MPS VII 47–49, but it failed to prevent the neurological deterioration and cognitive decline even when performed early in the disease course 50. This is no longer considered a viable treatment option 51. Although HSCT with bone marrow cells does not prevent the cognitive and neurological decline in MPS III, animal studies using stem cells from umbilical cord blood have shown more promise (reviewed in 52). This strategy is now being tested in a few patients with MPS IIIA and IIIB.

Intravenous ERT is currently available for MPS I, II and VI. Intravenously delivered ERT does not cross the blood–brain barrier and does not address the neurological and cognitive manifestations of the MPSs, making it ill-suited as a therapy for MPS III. Intrathecal ERT delivered to the brain via an implanted device has shown strong promise in animal models of MPS I 53 and is currently being tested in human MPS I, MPS II and MPS IIIA patients in ongoing phase I/II clinical trials (NCT00852358, NCT00638547, NCT00920647, NCT01506141 NCT01155778, NCT01299727).

Substrate reduction therapy in MPS III uses small molecules to inhibit the synthesis of GAGs, thereby reducing the amount of storage material. These molecules may be able to cross the blood–brain barrier 54. Miglustat, an approved substrate reduction therapy for the LSD Type 1 Gaucher disease, was not associated with any improvement/stabilization in behaviour problems or cognitive function in a phase 3 trial in MPS III 55. Treatment of MPS IIIB mice with a synthetic version of the soy isoflavone genistein, a putative substrate reduction therapy agent, has shown potential in animal studies, as complete correction of behaviour was reported in the mouse model of MPS IIIB 56. Soy isoflavone extracts have been tested in open-label pilot studies in human patients, with resulting moderate improvements in gastrointestinal symptoms, skin texture, hair morphology and the frequency of infections; however, the effects upon cognitive function and overall disability scores have been minimal 57,58. A recent double-blind, placebo-controlled, crossover study of a genistein-rich soy isoflavone extract (10 mg/kg/day of genistein) for patients with MPS III revealed a significant, albeit small, reduction in plasma heparan sulfate concentration and in uGAG excretion 59. No effect on behaviour was observed, and parents or caregivers could not determine during which period of the crossover a patient was on genistein. Higher doses of synthetic genistein may be more efficient than the lower doses of soy isoflavone extracts used in clinical studies to date; this awaits further study 60.

## Conclusion

MPS III is an inherited metabolic disorder with a heterogeneous presentation and a progressive clinical course characterized by progressive neurocognitive decline, behavioural difficulties and relatively mild somatic manifestations (Table 1). It is believed that the most benefit from therapies in development will be seen when treatment is begun before irreversible cognitive decline has occurred. Thus, early recognition and diagnosis are key to optimizing patient outcomes in this population, and paediatricians and other paediatric clinicians play a critical role. Because of the subtlety of the somatic features, young children with MPS III are easily misdiagnosed with idiopathic developmental or speech delay, ADHD or autism spectrum disorders. Clinicians must step back and view the whole child with awareness of the possibility of a metabolic disorder. The presence of developmental or speech delay comorbid with any characteristic somatic sign or symptom or with behavioural difficulties should prompt diagnostic testing (Fig. 2). The threshold for diagnostic testing can be low, especially since uGAG excretion assays are noninvasive and inexpensive, keeping in mind that a negative uGAG result *does not* rule out the disease. Those with a positive uGAG test or those for whom there is a high level of clinical suspicion should be tested with an enzyme activity assay, which can also determine the subtype. Molecular genetic testing and prenatal diagnosis can be offered to all families of MPS III patients in order to identify carriers and to facilitate informed family planning decisions. Once the diagnosis has been made, patients should be referred to a metabolic specialist, but the paediatrician/primary care physician retains a critical role in the multidisciplinary management team.

## Abbreviations

ADHD: Attention deficit/hyperactivity disorder
ERT: Enzyme replacement therapy
GAG: Glycosaminoglycan
HSCT: Hematopoietic stem cell transplantation
LSD: Lysosomal storage disease
MPS: Mucopolysaccharidosis
uGAG: Urinary glycosaminoglycan
